# Academic Practice or Primitive Physic

**Published:** 1987-05

**Authors:** D. G. Craig

**Affiliations:** Senior Lecturer, Dept. of General Practice, Guy's Hospital Medical School, London


### Academic Practice or Primitive Physic

**A review of a lecture given to the Society on 9.4.86 by Dr D. G. Craig MA. BM. BCh. (Oxf). F.R.C.G.P. DMJ. D.Obst. RCOG.**
Senior Lecturer, Dept. of General Practice, Guy's Hospital Medical School, London.

The lecturer brought fraternal greetings to the Society from the West Kent Medico-Chirurgical Society of South East London and the best wishes of Guy's Hospital Medical School to our emergent Department of General Practice.

He gave personal detail of the two halves of his professional life—firstly in a suburban general practice in the Midlands and more recently on the Plumstead and Erith marshes where the Guy's Department of General Practice focusses it's teaching output. Gallions Reach Health Centre, newly opened, is the site of an elegant Academic Unit destined to be the field unit for a programme of expansion into a general practice firm.

He outlined three activities of the present teaching programme:-

a) a structured experience of interviewing for pre-clinical students.
b) a personal study of referral diagnoses illustrating the G.P. hospital interface.
c) an example of the use of great literature as a teaching tool in small group work (1, 2, 3).

He reflected upon a recent visit to centres of excellence and experiment in the USA.

i) a look at the fashionable topic of medical problem solving emphasising that hypotheses are often limited and generated early and that competence is often case related (4).
ii) a run through of the findings of the Cambridge Conference on Medical Education which highlighted the opportunities that General Practice presents for 'advance organisation' of themes and concepts (5).
iii) an explanation of the Primary Care Curriculum as seen at Albuquerque—a synthesis of McMaster technology and early exposure to clinical material and topic teaching.
iv) an exposition of evaluation techniques seen in the University of New Mexico, where programmed patients, social scientists, check lists, one way mirrors and video cameras are put together in an encouraging format for young medical students.
v) the feeling that a Department of General Practice should take over The Introduction to Clinical Medicine Course: any that do have no need to justify their existence and there is no problem of credibility with the student body.

Taking Inglefinger's bon mot about the Primary Physician as 'A decision maker in the presence of uncertainty' as his theme the lecturer developed it in various ways.

a) the Dr Fox lecture where a bogus but extrovert lecturer delivered a lecture entitled 'Mathematical Game Theory as applied to Physician Education' to a series of sophisticated audiences. It seemed possible to obtain a high rating with no content but high energy (6).
b) Galton's 19th century article on the efficacy of prayer and its analysis of the post hoc propter hoc argument (7).
c) Rosenhams' work on pseudo patients and the effect of labelling and environment on diagnosis (8).
d) some simple work of his own on the varying criteria of normality in gynaecology.
e) a summary of Meador's work on non-disease—mimicking, over interpretation and the like (10).
f) Milgram's work on the effects of punishment on learning (11).

In the face of so many uncertainties he attempted to define a role for the Department of General Practice.

1.1 To encourage divergent thinking of both causation and significance of disease and treatment options.
1.2 To emphasise communication skills.
1.3 To introduce community medicine, at risk groups and the relevance of household interaction.
1.4 To discourage early intervention and decision making in the presence of inadequate evidence. To examine factors contributing to patient understanding and conformity. To experiment with non directive methods and simple counselling.
1.5 Prepare the student for the specialities—to give him a cool appraising eye for received wisdom.

In conclusion he read from Wesley's 18th century Home Doctor 'Primitive Physic' which besides a moderation derived from Sydenham's lectures extolled the virtues of wholemeal bread and bran (12).

Some quotations from the lecture for your day book.

Anon: "You get a better notion of the merits of the dinner from the guests than you do from the cook".
Audy J.R.: "Too much of a doctor's time is now taken up by the unhealthy people who are repeatedly getting ill" (13).
Dubos R: "Disease is an inevitable component of human life, so health as the absence of disease is an abstract and unattainable ideal" (14).
Gold, Greenlees and Trott: "We suspect the self evident".
Hoke R: "There is a healthy way to live a disease" (15).
Wesley J: "Nothing conduces more to health than abstinence and plain food with due labour" (12).

### REFERENCES

1. MOORE, (1978) The Missing Medical Text.

2. ROBERT GRAVES, Call it a Good Marriage.

3. VIRGINIA WOOLF, On Being Ill.

4. ELSTEIN, SHULMAN, SPRAFKA, (1978) Medical Problem Solving.

5. JOLLY, COLES *et al* (1976) Directions in Clinical Assessment.

6. NAFTULIN, WARE, DONNELLY (1973) Dr Fox Lecture: a paradigm of educational seduction *J.Med.Education* **48**, 630-635.

7. GALTON, (1872) Statistical Enquiries into the Efficacy of Prayer *Fortnightly Review* **48**, 126-135.

8. ROSENHAM, (1973) On Being Sane in Insane Places *Science* **1** 979, 250–258.

9. SCAMBLER, SCAMBLER, CRAIG (1981) Kinship and Friendship Networks and Women's Demands for Primary Care *JRCGP* **31**, 746-750.

10. MEADOR (1965) *New England Journal of Medicine* **272**, 92-95.

11. MILGRAM (1968) *International Journal of Psychiatry* **6**, 259-275.

12. WESLEY (1747) Primitive Physic.

13. AUDY (1970) *California Medicine* **113/5**, 48-53.

14. DUBOS (1965) Man Adapting.

15. HOKE (1974) *Ekistics* **220**, 169-172.

